# miR-203 Suppresses the Proliferation and Migration and Promotes the Apoptosis of Lung Cancer Cells by Targeting SRC

**DOI:** 10.1371/journal.pone.0105570

**Published:** 2014-08-20

**Authors:** Nan Wang, Hongwei Liang, Yong Zhou, Chen Wang, Suyang Zhang, Yi Pan, Yanbo Wang, Xin Yan, Junfeng Zhang, Chen-Yu Zhang, Ke Zen, Donghai Li, Xi Chen

**Affiliations:** 1 Jiangsu Engineering Research Center for microRNA Biology and Biotechnology, State Key Laboratory of Pharmaceutical Biotechnology, School of Life Sciences, Nanjing University, Nanjing, Jiangsu, China; 2 Department of Thoracic and Cardiovascular surgery, Affiliated Gulou Hospital, Medical college of Nanjing University, Nanjing, Jiangsu, China; 3 The Comprehensive Cancer Center of Drum Tower Hospital affiliated to Medical School of Nanjing University & Clinical Cancer Institute of Nanjing University, Nanjing, Jiangsu, China; H.Lee Moffitt Cancer Center & Research Institute, United States of America

## Abstract

SRC, also known as proto-oncogene c-Src, is a non-receptor tyrosine kinase that plays an important role in cancer progression by promoting survival, angiogenesis, proliferation, and invasion pathways. In this study, we found that SRC protein levels were consistently upregulated in lung cancer tissues, but that SRC mRNA levels varied randomly, suggesting that a post-transcriptional mechanism was involved in SRC regulation. Because microRNAs (miRNAs) are powerful post-transcriptional regulators of gene expression, we used bioinformatic analyses to search for miRNAs that potentially target SRC. We identified specific targeting sites for miR-203 in the 3′-untranslated region (3′-UTR) of SRC. We then experimentally validated miR-203 as a direct regulator of SRC using cell transfection and luciferase assays and showed that miR-203 inhibited SRC expression and consequently triggered suppression of the SRC/Ras/ERK pathway. Finally, we demonstrated that the repression of SRC by miR-203 suppressed the proliferation and migration and promoted the apoptosis of lung cancer cells. In summary, this study provides the first clues regarding the role of miR-203 as a tumor suppressor in lung cancer cells through the inhibition of SRC translation.

## Introduction

Lung cancer is the leading cause of cancer-related deaths worldwide, and non–small cell lung cancer (NSCLC) accounts for approximately 80% of all cases [1]. The majority of lung cancers (56%) are diagnosed at a distant stage because early disease is typically asymptomatic; only 15% of cases are diagnosed at a local stage [2]. Indeed, patients with lung cancer often exhibit tumor cell invasion and metastasis before diagnosis, which renders current treatments, including surgery, radiotherapy, and chemotherapy, ineffective. The overall 5-year survival rate for non-small cell lung cancer is extremely low (17.1%). Therefore, studying the molecular basis of lung cancer is crucial for designing new therapeutic agents that will improve the survival rate.

With the substantial advances in our understanding of tumor biology, key signaling pathways involved in mediating lung cancer growth and progression have been identified [3]. Dominant oncogenes and tumor suppressor genes involved in the pathogenesis of lung cancer have attracted substantial interest, and their central roles and fundamental contribution to the misbehavior of cancer cells have become clear [4]. These genes offer new targets for biological therapies. One such target is SRC (also known as c-Src), a proto-oncogene encoding a tyrosine kinase that is frequently overexpressed and activated in many cancer types, including lung cancer [5], [6]. On the molecular basis, SRC regulates multiple signaling cascades associated with tumor development and progression, including the focal adhesion kinase (FAK) pathway, the epidermal growth factor receptor (EGFR) pathway, and the Ras/ERK pathway [7]. Consequently, SRC functions as an oncogene to favor proliferation, migration, and invasion of various types of cancer cells [8], [9]. Despite these recent advances in our understanding of the important roles of SRC in tumorigenesis, the precise molecular mechanism through which SRC contributes to lung cancer progression remains to be fully elucidated.

A class of small, non-coding, single-stranded RNAs known as microRNAs (miRNAs) has been shown to be involved in cancer. miRNAs bind target mRNAs at complementary sites in their 3′-untranslated regions (3′-UTRs), thereby suppressing the expression of the target gene at the posttranscriptional level [10], [11]. Through this mechanism, miRNAs regulate a wide range of biological processes, including cell proliferation and differentiation, migration, apoptosis, development, and metabolism [10]. On the other hand, dysfunction of miRNAs is implicated in various human cancers, including lung cancer, and miRNAs can function as both oncogenes and tumor suppressors during carcinogenesis [12], [13]. For example, low expression of let-7a and high expression of the miR-17-92 cluster are associated with a poor clinical outcome in lung cancer [14], [15]. Moreover, it was reported that miR-31 could directly repress the tumor suppressors LATS2 and PPP2R2A in human lung cancer [16]. These findings underscore the need for an in-depth search for miRNAs that are aberrantly expressed during lung carcinogenesis as well as the need for an intensive investigation of their role in tumor biology.

Although the deregulation of miRNAs and SRC play important roles in lung carcinogenesis, no correlation between SRC and miRNAs in lung cancer has been reported. In this study, we predicted that SRC is a target of miR-203. After measuring the expression levels of miR-203 and SRC in human lung cancer tissue and paired noncancerous tissue samples, we detected an inverse correlation between miR-203 and SRC protein levels. Furthermore, we experimentally validated the direct inhibition of SRC translation by miR-203 using cell transfection and luciferase assays. Finally, we showed that miR-203 inhibited SRC expression and consequently triggered the suppression of the downstream signaling pathways of SRC, such as the SRC/Ras/ERK pathway, which eventually suppressed the proliferation and migration and promoted the apoptosis of lung cancer cells.

## Materials and Methods

### Cells and human tissues

The human lung cancer cell lines A549, HCC827, and H1975, and the human normal lung fibroblast cell line HLF were purchased from the Shanghai Institute of Cell Biology, Chinese Academy of Sciences (Shanghai, China). A549, HCC827, and H1975 cells were cultured in DMEM supplemented with 10% fetal bovine serum (GIBCO, CA, USA), and HLF cells were cultured in F12K supplemented with 10% fetal bovine serum (GIBCO) and 2.5 g/L NaHCO_3_. All cells were incubated in a 5% CO_2_ at 37°C in a water-saturated atmosphere. The lung tumors and paired normal adjacent tissues were derived from patients undergoing a surgical procedure at the Affiliated Gulou Hospital of Nanjing University (Nanjing, China). Then, tumor section slides were subjected to immunohistochemical analysis of SRC (sc-8056, Santa Cruz Biotechnology, CA, USA) according to the manufacturer's instructions. All of the patients or their guardians provided written consent, and the Ethics Committee from Nanjing University approved all aspects of this study. Tissue fragments were immediately frozen in liquid nitrogen at the time of surgery and stored at −80°C. The clinical features of the patients are listed in Table S1 in File S1.

### RNA isolation and quantitative RT-PCR

Total RNA was extracted from the cultured cells and human tissues using TRIzol Reagent (Invitrogen, Carlsbad, CA) according to the manufacturer's instructions. Assays to quantify miRNAs were performed using Taqman miRNA probes (Applied Biosystems, Foster City, CA) according to the manufacturer's instructions. Briefly, 1 µg of total RNA was reverse-transcribed to cDNA using AMV reverse transcriptase (TaKaRa, Dalian, China) and a stem-loop RT primer (Applied Biosystems). The reaction conditions were as follows: 16°C for 30 min, 42°C for 30 min, and 85°C for 5 min. Real-time PCR was performed using a TaqMan PCR kit on an Applied Biosystems 7300 Sequence Detection System (Applied Biosystems). The reactions were incubated in a 96-well optical plate at 95°C for 10 min, followed by 40 cycles of 95°C for 15 s and 60°C for 1 min. All of the reactions were run in triplicate. After the reaction, the cycle threshold (C_T_) data were determined using fixed threshold settings, and the mean C_T_ of the triplicate PCRs was determined. A comparative C_T_ method was used to compare each condition to the controls. The relative levels of the miRNAs in cells and tissues were normalized to U6. The amount of miRNA relative to the internal control U6 was calculated using the 2^−ΔΔCT^ equation, in which ΔΔC_T_  =  (C_T miRNA_ - C_T U6_)_target_ - (C_T miRNA_ - C_T U6_)_control_. To quantify the SRC mRNA, 1 µg of total RNA was reverse-transcribed to cDNA using oligo dT and Thermoscript (TaKaRa) in the reaction, which was performed with the following conditions: 42°C for 60 min and 70°C for 10 min. Next, real-time PCR was performed using the RT product, SYBER Green Dye (Invitrogen) and specific primers for SRC and GAPDH. The sequences of the primers were as follows: SRC (sense): 5′-CATCCAAGCCTCAGACCCA-3′; SRC (antisense): 5′-TGACACCACGGCATA CAGC-3′; GAPDH (sense): 5′-GATATTGTTGCCATCAATGAC-3′; and GAPDH (antisense): 5′-TTGATTTTGGAGGGATCTCG-3′. The reactions were incubated at 95°C for 5 min, followed by 40 cycles of 95°C for 30 s, 60°C for 30 s, and 72°C for 30 s. After the reactions were complete, the C_T_ values were determined by setting a fixed threshold. The relative amount of SRC mRNA was normalized to GAPDH.

### Overexpression and knockdown of miR-203

Synthetic pre-miR-203, anti-miR-203 and scrambled negative control RNAs were purchased from Ambion (Austin, TX, USA). All cells were seeded in 6-well plates or 60-mm dishes, and the cells were transfected with Lipofectamine 2000 (Invitrogen) the following day when the cells were approximately 70% confluent. In each well, equal amounts of pre-miR-203, anti-miR-203 or scrambled negative control RNA were used. The cells were harvested 24 h after transfection for quantitative RT-PCR and Western blotting.

### Luciferase reporter assay

To test the direct binding of miR-203 to the target gene SRC, a luciferase reporter assay was performed as previously described [17]. The entire 3′-untranslated region (3′-UTR) of human SRC was amplified using PCR with human genomic DNA as a template. The PCR products were inserted into the p-MIR-reporter plasmid (Ambion), and the insertion was confirmed as correct by sequencing. To test the binding specificity, the sequences that interacted with the miR-203 seed sequence were mutated (from AUUUCA to UAAAGU), and the mutant SRC 3′-UTR was inserted into an equivalent luciferase reporter. For luciferase reporter assays, A549 cells were cultured in 24-well plates, and each well was transfected with 1 µg of firefly luciferase reporter plasmid, 1 µg of a β-galactosidase (β-gal) expression plasmid (Ambion), and equal amounts (100 pmol) of pre-miR-203, anti-miR-203 or the scrambled negative control RNA using Lipofectamine 2000 (Invitrogen). The β-gal plasmid was used as a transfection control. Twenty-four hours post-transfection, the cells were assayed using a luciferase assay kit (Promega, Madison, WI, USA).

### Plasmid construction and siRNA interference assay

An siRNA sequence targeting the human SRC cDNA was designed and synthesized by GenePharma (Shanghai, China). The siRNA sequence was 5′-CACUACAAGAUCCGGAAAC-3′. A scrambled siRNA was included as a negative control. A mammalian expression plasmid encoding the human SRC open reading frame (pReceiver-M02-SRC) was purchased from GeneCopoeia (Germantown, MD, USA). An empty plasmid served as a negative control. The SRC expression vector and SRC siRNA were transfected into A549 cells using Lipofectamine 2000 (Invitrogen) according to the manufacturer's instructions. Total RNA and protein were isolated 24 h post-transfection. The SRC mRNA and protein expression levels were assessed by quantitative RT-PCR and Western blotting.

### Protein extraction and Western blotting

All cells were rinsed with PBS (pH 7.4) and lysed in RIPA Lysis buffer (Beyotime, China) supplemented with a Protease and Phosphatase Inhibitor Cocktail (Thermo Scientific 78440) on ice for 30 min. The tissue samples were frozen solid with liquid nitrogen, ground into a powder and lysed in RIPA Lysis buffer containing the Protease and Phosphatase Inhibitor Cocktail on ice for 30 min. When necessary, sonication was used to facilitate lysis. Cell lysates or tissue homogenates were centrifuged for 10 min (12000 g, 4°C). The supernatant was collected, and the protein concentration was calculated using a Pierce BCA protein assay kit (Thermo Scientific, Rockford, IL, USA). The protein levels were analyzed using western blots with corresponding antibodies. The protein levels were normalized by probing the same blots with a GAPDH antibody. The antibodies were purchased from the following sources: anti-c-Src (B-12) (sc-8056, Santa Cruz Biotechnology), anti-ERK1/2 (pT202/pY204) (BD Biosciences 612359, USA), anti-ERK1 (BD Biosciences 610031, USA), anti-PKCalpha (H-7) (sc-8393, Santa Cruz Biotechnology) and anti-GAPDH (sc-365062, Santa Cruz Biotechnology). Ras activity was detected using the Ras Activation Assay Kit from Upstate-Millipore (17–218). Protein bands were analyzed using the ImageJ software.

### Cell viability assay

To assess cell viability, A549 cells were seeded in triplicate in 96-well plates at a density of 5×10^3^ cells per well in 100 µL of culture medium. The cell proliferation index was measured using the 3-(4,5-dimethylthiazol-2-yl)-2,5-diphenyl tetrazolium bromide (MTT) assay (Sigma, USA), which was performed 12, 24, 36, and 48 h after transfection according to the manufacturer's instructions.

### Cell migration assay

The migration ability of A549 cells transfected with the miR-203 mimic or SRC overexpression plasmid was tested in a Transwell Boyden Chamber (6.5-mm, Costar, USA). The polycarbonate membranes (8- µm pore size) on the bottom of the upper compartment of the Transwells were coated with 1% human fibronectin (R&D systems 1918-FN, USA). The cells were harvested 24 h after transfection and suspended in FBS-free DMEM culture medium. Then, cells were added to the upper chamber (4×10^4^ cells/well). At the same time, 0.5 mL of DMEM with 10% FBS was added to the lower compartment, and the Transwell-containing plates were incubated for 12 h in a 5% CO_2_ atmosphere saturated with H_2_O. After incubation, cells that had entered the lower surface of the filter membrane were fixed with 4% paraformaldehyde for 25 min at room temperature, washed 3 times with distilled water and stained with 0.1% crystal violet in 0.1 M borate and 2% ethanol for 15 min at room temperature. Cells remaining on the upper surface of the filter membrane (non-migrant) were scraped off gently with a cotton swab. Images of the lower surfaces (with migrant cells) were captured by a photomicroscope (5 fields per chamber) (BX51 Olympus, Japan), and the cells were counted blindly.

### Apoptosis assays

The apoptosis of A549 cells transfected with the miR-203 mimic or SRC overexpression plasmid was tested using an Annexin V-FITC/propidium iodide (PI) staining assay. A549 cells were cultured in 12-well plates and transfected with miR-203 mimic, siRNA, or the SRC overexpression plasmid to induce apoptosis. Pre-miR-control and control siRNAs served as negative controls. Cells were cultured overnight with both serum-containing complete medium and serum-depleted medium; and the attached and floating cells were then harvested. Flow cytometry analysis of apoptotic cells was carried out using an Annexin V-FITC/PI staining kit (BD Biosciences, CA, USA). After washes with cold PBS, the cells were resuspended in binding buffer (100 mM HEPES, pH 7.4, 100 mM NaCl, and 25 mM CaCl_2_) followed by staining with Annexin V-FITC/PI at room temperature in darkness for 15 min. Apoptotic cells were then evaluated by gating PI and Annexin V-positive cells on a fluorescence-activated cell-sorting (FACS) flow cytometer (BD Biosciences, San Jose, CA). All experiments were performed in triplicate.

### Statistical analysis

All of the images of Western blotting are representative of at least three independent experiments. Quantitative RT-PCR, the luciferase reporter assay, and the cell viability and apoptosis assays were performed in triplicate, and each experiment was repeated several times. The data shown are the mean ± SE of at least three independent experiments. The differences were considered statistically significant at p<0.05 using Student's t -test.

## Results

### Upregulation of the SRC protein, but not mRNA, in lung cancer tissues

**Figure 1:**
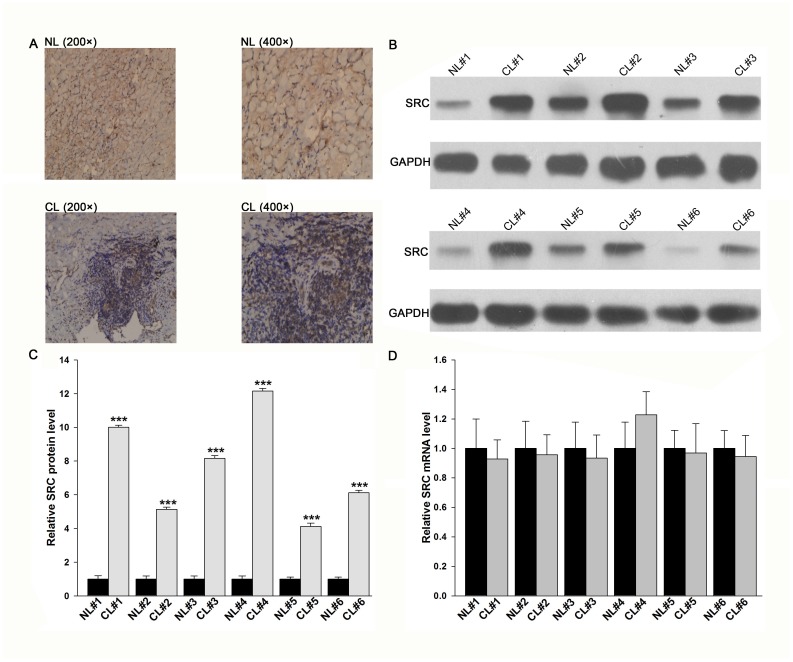
Expression levels of the SRC protein and mRNA in lung cancer tissues. (**A**) Representative image of immunohistochemical analysis of SRC expression in lung cancer (CL) and normal adjacent tissue (NL) samples. (**B and C**) Western blotting analysis of the expression levels of the SRC protein in six pairs of CL and NL samples. B: representative image; C: quantitative analysis. (**D**) Quantitative RT-PCR analysis of the relative expression levels of SRC mRNA in six pairs of CL and NL samples. * P<0.05; ** P<0.01.

We first investigated SRC expression by means of both immunohistochemical analysis and immunoblot analysis in human lung cancer tissues and corresponding noncancerous tissues. We found that SRC protein levels were dramatically higher in the lung cancer tissues (Figure 1, A–C). Although the SRC protein was consistently upregulated in lung cancer tissues, SRC mRNA levels did not differ significantly between the cancer and noncancerous tissues (Figure 1D). Furthermore, we found that the SRC protein level was higher in human lung adenocarcinoma A549 cells compared to that in normal lung fibroblast HLF cells (Figure S1, A and B in File S1), but the SRC mRNA level was equal in these two cell lines (Figure S1C in File S1). This disparity between protein and mRNA in SRC expression in lung cancers strongly suggests that a post-transcriptional mechanism is involved in SRC regulation.

### Identification of conserved miR-203 target sites within the 3′-UTR of SRC

**Figure 2:**
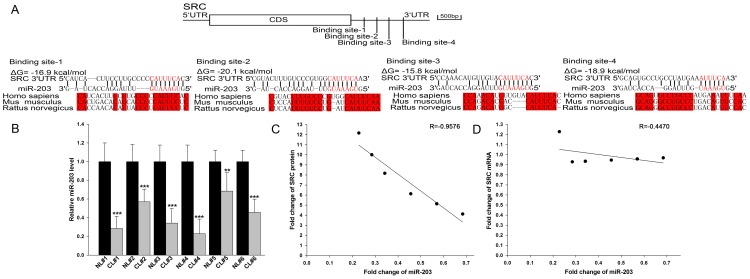
Detection of an inverse correlation between the miR-203 and SRC levels in lung cancer tissue samples. (**A**) Schematic description of the hypothetical duplexes formed by the interactions between the binding sites in the SRC 3′-UTR (top) and miR-203 (bottom). The predicted free energy value of each hybrid is indicated. The seed recognition sites are denoted, and all nucleotides in these regions are highly conserved across species, including human, mouse, and rat. (**B**) Quantitative RT-PCR analysis of the expression levels of miR-203 (in the form of the miRNA/U6 ratio) in six pairs of CL and NL samples. (**C**) Pearson's correlation scatter plot of the fold change of the levels of miR-203 and SRC protein in human lung cancer tissues. (**D**) Pearson's correlation scatter plot of the fold change of the levels of miR-203 and SRC mRNA in human lung cancer tissues. * P<0.05; ** P<0.01.

One important mode of post-transcriptional regulation is the repression of mRNA transcripts by miRNAs. miRNAs are, therefore, likely to play a biologically relevant role in regulating SRC expression in lung cancer. Three computational algorithms, including TargetScan [18], miRanda [19], and PicTar [20], were used in combination to identify potential miRNAs that can target SRC. Using these approaches, miR-203 was identified as a candidate regulatory miRNA of SRC. The predicted interaction between miR-203 and the target sites in the SRC 3′-UTR are illustrated in Figure 2A. Four potential miR-203 target sites were found in the 3′-UTR of the SRC mRNA sequence. The minimum free energy values of these hybrids were −16.9, −20.1, −15.8, and −18.9 kcal/mol, respectively, which are well within the range of genuine miRNA-target pairs. Moreover, perfect base pairing occurred between the seed region (the core sequence that encompasses the first 2–8 bases of the mature miRNA) and the cognate targets. Furthermore, the miR-203 binding sequences in the SRC 3′-UTR are highly conserved across species.

### Detection of an inverse correlation between the miR-203 and SRC levels in lung cancer tissue samples

miRNAs are generally thought to have expression patterns that are opposite to that of their targets [21]–[23]. We next investigated whether miR-203 was inversely correlated with SRC in lung cancer. After determining the levels of miR-203 in the same six pairs of lung cancer tissues and the corresponding noncancerous tissues, we showed that the miR-203 levels were consistently downregulated in lung cancer tissues (Figure 2B). The inverse correlation between miR-203 levels and SRC protein levels (Figure 2C) and the disparity between miR-203 levels and SRC mRNA levels (Figure 2D) were further illustrated using Pearson's correlation scatter plots. As observed, an inverse correlation between the miR-203 levels and SRC protein levels, but not mRNA levels, was observed in human lung cancer tissues. Likewise, miR-203 level was also lower in A549 cells compared to that in HLF cells (Figure S1D in File S1). The results strongly indicated that a typical, miRNA-mediated, post-transcriptional regulation mechanism was involved in SRC repression.

### Validation of SRC as a direct target of miR-203

**Figure 3:**
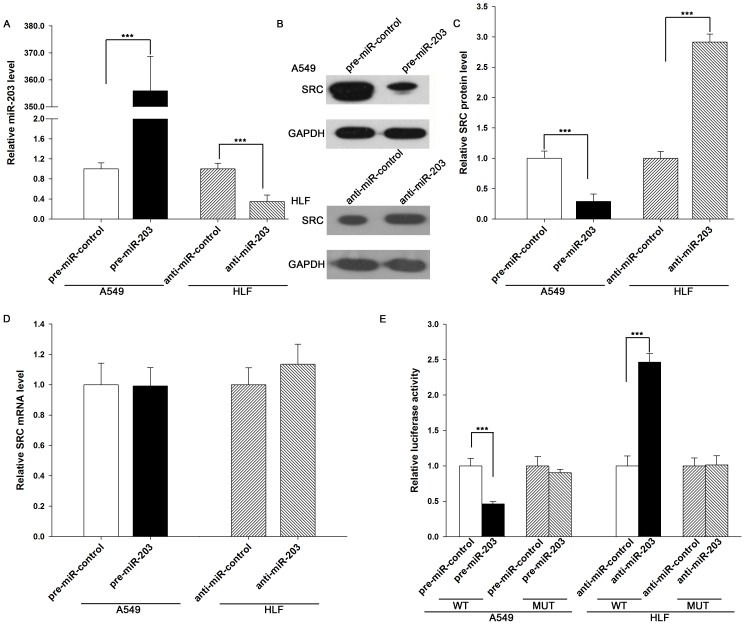
Direct regulation of SRC expression by miR-203 at the posttranscriptional level. (**A**) Quantitative RT-PCR analysis of the miR-203 levels in A549 cells treated with pre-miR-control or pre-miR-203 and in HLF cells treated with anti-miR-control or anti-miR-203. (**B and C**) Western blot analysis of SRC protein levels in A549 cells treated with pre-miR-control or pre-miR-203 and in HLF cells treated with anti-miR-control or anti-miR-203. B: representative image; C: quantitative analysis. (**D**) Quantitative RT-PCR analysis of SRC mRNA levels in A549 cells treated with pre-miR-control or pre-miR-203 and in HLF cells treated with anti-miR-control or anti-miR-203. (**E**) Firefly luciferase reporters containing wild-type (WT) or mutant (MUT) miR-203 binding sites in the SRC 3′-UTR were co-transfected into A549 cells with pre-miR-control or pre-miR-203 and into HLF cells with anti-miR-control or anti-miR-203. Twenty-four hours post-transfection, the cells were assayed using a luciferase assay kit. The results are calculated as the ratio of firefly luciferase activity in the pre-miR-203- or anti-miR-203-transfected cells normalized to the control cells. * P<0.05; ** P<0.01.

The correlation between miR-203 and SRC was further examined by evaluating SRC expression in human lung adenocarcinoma A549 cells after overexpression of miR-203. In these experiments, miR-203 overexpression was achieved by transfecting the cells with pre-miR-203 (a synthetic RNA oligonucleotide duplex mimicking the miR-203 precursor). As anticipated, miR-203 levels in A549 cells were increased more than 300-fold when these cells were transfected with pre-miR-203 (Figure 3A). To validate that transfection of miR-203 was successful, one of the representative target genes of miR-203, PKCalpha (REF), was assessed and it was shown that PKCalpha was significantly downregulated in A549 cells after transfection of miR-203 (Figure S2 in File S1). Likewise, the expression of the SRC protein was reduced by the introduction of miR-203 in A549 cells (Figure 3, B and C). To determine the level at which miR-203 influenced SRC expression, we repeated the above-mentioned experiments and examined the expression of SRC mRNA after transfection. Although the intracellular level of miR-203 was significantly altered after treatment with pre-miR-203, the overexpression of miR-203 did not affect SRC mRNA stability (Figure 3D). On the other hand, the correlation between miR-203 and SRC was also examined by evaluating SRC expression in normal lung fibroblast HLF cells after knockdown of miR-203 with anti-miR-203 (chemically modified antisense oligonucleotides designed to specifically target mature miR-203) (Figure 3A). Knocking down miR-203 in HLF cells resulted in the increase of SRC protein (Figure 3, B and C) but not mRNA (Figure 3D) levels. These results demonstrate that miR-203 specifically regulates SRC protein expression at the post-transcriptional level, which is a typical animal miRNA-mediated regulation mechanism. To demonstrate the robustness of the test, we repeated the above-mentioned experiments in two additional lung adenocarcinoma cell lines, HCC827 and H1975. Likewise, the miR-203 levels were significantly increased when HCC827 and H1975 cells were transfected with pre-miR-203 (Figure S3, A and E in File S1). Consequently, the SRC protein levels were suppressed when HCC827 and H1975 cells were treated with pre-miR-203 (Figure S3, B, C, F and G in File S1), but the SRC mRNA levels were not influenced by such treatment (Figure S3, D and H in File S1).

To determine whether the negative regulatory effects that miR-203 exerted on SRC expression were mediated through the binding of miR-203 to the presumed sites in the 3′-UTR of the SRC mRNA, the full length SRC 3′-UTR containing the four presumed miR-203 binding sites was fused downstream of the firefly luciferase gene in a reporter plasmid. The resulting plasmid was transfected into A549 cells along with a transfection control plasmid (β-gal) and pre-miR-203. As expected, the overexpression of miR-203 resulted in a more than 50% reduction of luciferase reporter activity compared with cells treated with the negative control RNA (Figure 3E). Furthermore, we introduced point mutations into the corresponding complementary sites in the SRC 3′-UTR to eliminate the predicted miR-203 binding sites (all of the four binding positions were mutant). This mutated luciferase reporter was unaffected by the overexpression of miR-203 (Figure 3E). In contrast, when the resulting plasmid was transfected into HLF cells along with a transfection control plasmid (β-gal) and anti-miR-203, the knockdown of miR-203 resulted in a more than 75% increase of luciferase reporter activity compared with cells treated with the negative control RNA, while the mutated luciferase reporter was unaffected by the knockdown of miR-203 (Figure 3E). This finding suggests that the binding sites strongly contribute to the miRNA:mRNA interaction mediating the post-transcriptional repression of SRC expression. In conclusion, our results demonstrate that miR-203 directly recognizes and binds to the 3′-UTR of the SRC mRNA transcript and inhibits SRC translation.

### Suppression of the SRC/Ras/ERK signaling pathway by miR-203

**Figure 4:**
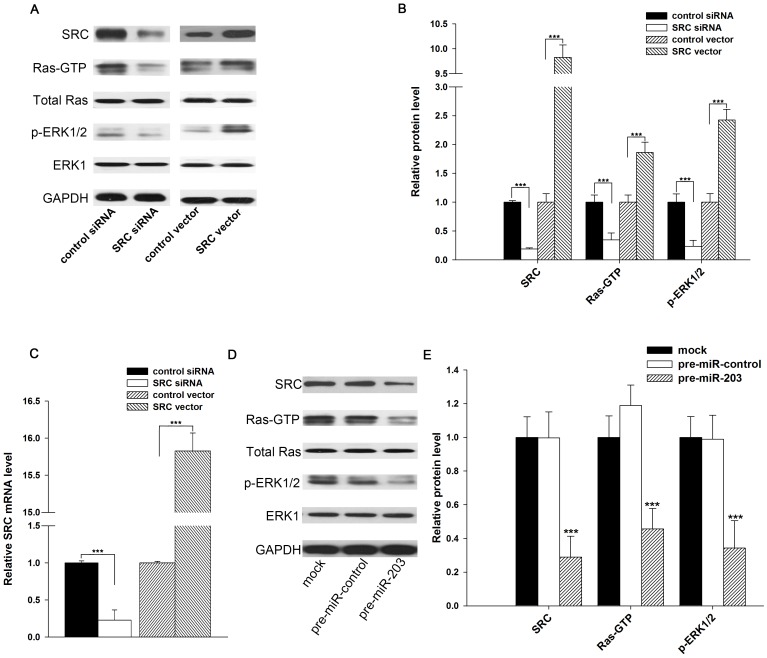
miR-203 represses the SRC/Ras/ERK signal pathway by silencing SRC. (**A and B**) Western blotting analysis of the protein levels of SRC, Ras-GTP, total Ras, phosphorylated ERK1/2, and ERK1 in A549 cells transfected with control siRNA, SRC siRNA, control vector, or SRC vector. A: representative image; B: quantitative analysis. (**C**) Quantitative RT-PCR analysis of the SRC mRNA levels in A549 cells treated with control siRNA, SRC siRNA, control vector, or SRC vector. (**D and E**) Western blotting analysis of the protein levels of SRC, Ras-GTP, total Ras, phosphorylated ERK1/2, and ERK1 in A549 cells transfected with pre-miR-control or pre-miR-203. D: representative image; E: quantitative analysis. * P<0.05; ** P<0.01.

We next analyzed the biological consequences of the decreased SRC expression caused by miR-203 in lung cancer cells. As a potent oncogene, SRC regulates the proliferation and motility of cancer cells by affecting the FAK, EGFR, and Ras/ERK signaling pathways [7]. Therefore, we investigated whether the suppression of SRC expression due to miR-203 regulates the activities of the molecules in the SRC/Ras/ERK signaling pathway. First, we validated the effect of SRC activity on the Ras/ERK signal pathway in A549 cells. In agreement with previous reports [7], [24], knockdown of SRC by siRNA-mediated RNA interference resulted in decreased SRC mRNA and protein levels (Figure 4, A–C), which, in turn, led to decreased Ras-GTP and phosphorylated-ERK1/2 (Figure 4, A and B). In contrast, overexpression of SRC resulted in increased SRC mRNA and protein levels (Figure 4, A–C), which, in turn, led to increased Ras-GTP and phosphorylated-ERK1/2 (Figure 4, A and B). Next, we investigated if miR-203 induction could mimic the SRC reduction in suppressing Ras/ERK signaling pathway. As anticipated, overexpression of miR-203 in A549 cells downregulated the SRC protein and resulted in less active Ras-GTP, which, in turn, led to less phosphorylated ERK1/2 (Figure 4, D and E). Taken together, the results suggest that Ras/ERK, as the downstream signaling pathway of SRC, may be tightly controlled by the miR-203/SRC regulatory pathway.

### The role of miR-203 in regulating SRC in lung cancer cells

**Figure 5:**
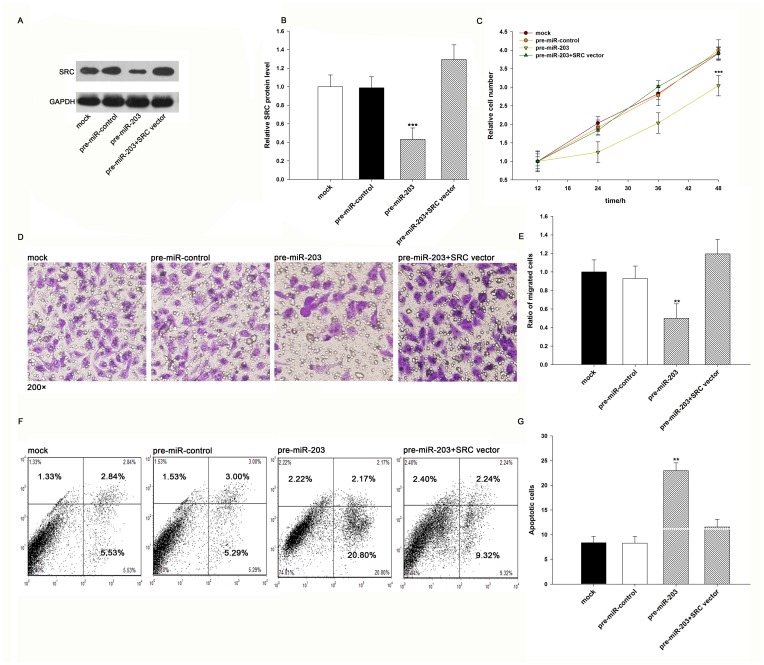
The role of miR-203 targeting SRC in the regulation of proliferation, migration, and apoptosis of lung cancer cells. (**A and B**) Western blotting analysis of the protein levels of SRC in A549 cells transfected with pre-miR-control, pre-miR-203, or pre-miR-203 plus the SRC-overexpressing vector. A: representative image; B: quantitative analysis. (**C**) The MTT viability assay was performed 12, 24, 36 and 48 h after the transfection of A549 cells with pre-miR-control, pre-miR-203, or pre-miR-203 plus the SRC-overexpressing vector. (**D and E**) Transwell analysis of A549 cells treated with equal doses of pre-miR-control, pre-miR-203, or pre-miR-203 plus the SRC-overexpressing vector. D: representative image; E: quantitative analysis. (**F and G**) A549 cells were transfected with equal doses of pre-miR-control, pre-miR-203, or pre-miR-203 plus the SRC-overexpressing vector. Cell apoptosis profiles were analyzed by flow cytometry. The biparametric histogram shows cells in early (bottom right quadrant) and late apoptotic states (upper right quadrant). Viable cells are double negative (bottom left quadrant). F: representative image; G: quantitative analysis. * P<0.05; ** P<0.01.

We next focused on studying the role of miR-203 in regulating SRC. Because SRC is known to be involved in the regulation of cell proliferation, apoptosis, and migration, we investigated whether miR-203 would regulate SRC to modulate cell proliferation, apoptosis, and migration in A549 cells. In support of the notion that SRC is essential in promoting proliferation [25], A549 cells transfected with SRC siRNA showed inhibition of cell proliferation (Figure S4A in File S1); in contrast, transfection with the SRC-overexpressing plasmid, which specially expresses the full-length open reading frame (ORF) of SRC without the miR-203–responsive 3′-UTR, had an opposite effect on cell proliferation (Figure S4B in File S1). Subsequently, we assessed the role of miR-203 on cell proliferation. As expected, A549 cells transfected with pre-miR-203 had decreased SRC protein levels (Figure 5, A and B) and proliferated at a significantly lower rate (Figure 5C). Moreover, compared to cells transfected with pre-miR-203, those transfected with pre-miR-203 and the SRC-overexpressing plasmid had recovered SRC protein levels (Figure 5, A and B) and exhibited significantly higher proliferation rates (Figure 5C), suggesting that miR-203-resistant SRC is sufficient to rescue the suppression of SRC by miR-203 and attenuate the anti-proliferative effect of miR-203 on lung cancer cells. Taken together, the results indicate that miR-203 might inhibit cell proliferation by silencing SRC.

Next, we assessed the effect of miR-203 and SRC on the migration ability of A549 cells. Transfection of SRC siRNA remarkably reduced the number of A549 cells that passed through the Transwell chamber, whereas transfection of the SRC-overexpressing plasmid increased the migration rate (Figure S4, C and D in File S1). Additionally, the percentage of migrated cells was significantly lower in A549 cells transfected with pre-miR-203 (Figure 5, D and E). However, when A549 cells were co-transfected with pre-miR-203 and the SRC-overexpressing plasmid, SRC dramatically attenuated the anti-migration effect of miR-203 (Figure 5, D and E). The results indicate that miR-203 might inhibit cell migration by silencing SRC.

Finally, we investigated cell apoptosis using flow cytometry analysis. In apoptosis assay, apoptosis was induced by serum starvation in the culture media (Figure S5 in File S1). Transfection of SRC siRNA remarkably increased the percentage of apoptotic cells when compared to cells transfected with control siRNA, whereas transfection of the SRC-overexpressing plasmid decreased cell apoptosis (Figure S4, E and F in File S1). In addition, significantly more apoptotic cells were observed in A549 cells transfected with pre-miR-203 compared with those transfected with the pre-miR-control (Figure 5, F and G). Furthermore, when A549 cells were simultaneously transfected with pre-miR-203 and the SRC-overexpressing plasmid, SRC dramatically attenuated the pro-apoptotic effect of miR-203 (Figure 5, F and G). Taken together, these results indicate that miR-203 might modulate cell apoptosis by downregulating SRC in lung cancer cells.

## Discussion

The cellular tyrosine kinase, SRC, is frequently overexpressed or aberrantly activated in a range of cancers [26]. For example, the increased expression of SRC has been reported in 60–80% of adenocarcinomas and 50% of squamous cell carcinomas from lung cancer patients [27]. Additionally, high levels of SRC activity have been reported in lung cancer, particularly adenocarcinomas, and the degree of kinase activity correlates with tumor size [28]. SRC can interact with many genetic and signaling pathways, including the FAK, EGFR, Ras/ERK, JAK/STAT, PI3K/AKT, and VEGF pathways [8], [26], [29]. These diverse substrate interactions link SRC with a broad range of oncogenic mechanisms, including cell proliferation, migration, apoptosis, and angiogenesis [8]. In this study, we showed that silencing SRC expression using siRNA could inhibit cell proliferation and migration and promote apoptosis in lung cancer cells, while overexpressing SRC had opposite effects, validating its role as an essential oncogene during lung tumorigenesis. Interestingly, we identified discordance between the SRC protein and mRNA levels in human lung cancer tissue samples and cultured cells. The results suggest a post-transcriptional regulation mechanism involved in SRC repression. One centrally important mode of post-transcriptional regulation is the repression of mRNA transcripts by miRNAs (25). Therefore, we searched for miRNAs that could target SRC, and experimentally validated miR-203 as a direct regulator of SRC. Interesting, SRC has been reported as a target gene of miR-203 in bladder cancer [30]. Taken together, our results identified a miRNA as a link between the SRC regulatory pathway and the pathogenesis of lung cancer.

In this study, we further showed that ectopic expression of miR-203 could inhibit the translation of SRC in lung cancer cells, which, in turn, was associated with a reduction in the levels of Ras-GTP and phosphorylated-ERK1/2. Thus, Ras/ERK signaling is also regulated by the miR-203/SRC regulatory pathway. The results reveal a new possible target for regulating Ras/ERK activity in lung cancer, which can be accomplished by affecting the miR-203 level. As the downstream signal transduction pathway of SRC, activation of Ras/ERK pathway is associated with cell transformation and tumor progression [31]. Consistent with this, we lastly showed that miR-203 could suppress SRC expression and, in turn, inhibit proliferation and migration and promote apoptosis of lung cancer cells. The results reveal a critical role for miR-203 as a tumor suppressor in lung carcinogenesis through the repression of SRC translation. Actually, miR-203 has been reported to act as a tumor-suppressive miRNA in many cancer types (30–34), and it was shown that miR-203 expression was downregulated in various prostate cancer cell lines [30]. The ectopic expression of miR-203 in prostate cancer cell lines could influence proliferation, apoptosis, and migration [30], [32], and the overexpression of miR-203 in laryngeal carcinoma cells reduced cell viability and led to a cell cycle arrest in G1 phase [33]. Additionally, expression of miR-203 suppressed cell proliferation and migration in human triple-negative breast cancer cells [34]. These studies, in summary, reveal the essential role of miR-203 as a tumor suppressor gene. Interestingly, it is noted that restoration of SRC expression can successfully attenuate the anti-proliferative, anti-migration, and pro-apoptotic effects of miR-203 on lung cancer cells, although miR-203 has many other targets. The results suggest that targeting SRC is a major mechanism by which miR-203 exerts its tumor-suppressive function. Therefore, the modulation of SRC by miR-203 might explain, at least in part, why the downregulation of miR-203 during lung carcinogenesis can accelerate lung cancer progression.

Taken together, this study delineates a novel regulatory network employing miR-203, SRC, and downstream signaling factors to fine-tune cell proliferation, migration, and apoptosis in lung cancer cells. This study may provide a potential novel target for future lung cancer therapy.

## Supporting Information

File S1 **Table S1, Figure S1–S5.** Table S1. Clinical features of lung cancer patients; Figure S1. the expression level of SRC and miR-203 in normal lung fibroblast cell line HLF cells and lung cancer cell lines A549 cells; Figure S2. miR-203 regulates PKCalpha expression in A549 cells; Figure S3. miR-203 directly regulates SRC expression at the post-transcriptional level; Figure S4. The role of SRC in the regulation of proliferation, migration, and apoptosis of lung cancer cells; Figure S5. The apoptosis of full-serum cultured A549 cells and serum-starvation cultured A549 cells.
(DOC)

## References

[1] Ramalingam S, Pawlish K, Gadgeel S, Demers R, Kalemkerian GP (1998) Lung cancer in young patients: analysis of a Surveillance, Epidemiology, and End Results database. J Clin Oncol 16: 651–657. 9469354 10.1200/JCO.1998.16.2.651

[2] Siegel R, DeSantis C, Virgo K, Stein K, Mariotto A, et al (2012) Cancer treatment and survivorship statistics, 2012. CA Cancer J Clin 62: 220–241. 22700443 10.3322/caac.21149

[3] Hanahan D, Weinberg RA (2011) Hallmarks of cancer: the next generation. Cell 144: 646–674. 21376230 10.1016/j.cell.2011.02.013

[4] Zochbauer-Muller S, Gazdar AF, Minna JD (2002) Molecular pathogenesis of lung cancer. Annu Rev Physiol 64: 681–708. 11826285 10.1146/annurev.physiol.64.081501.155828

[5] Johnson FM, Gallick GE (2007) SRC family nonreceptor tyrosine kinases as molecular targets for cancer therapy. Anticancer Agents Med Chem 7: 651–659. 18045060 10.2174/187152007784111278

[6] Rothschild SI, Gautschi O, Haura EB, Johnson FM (2010) Src inhibitors in lung cancer: current status and future directions. Clin Lung Cancer 11: 238–242. 20630825 10.3816/CLC.2010.n.030

[7] Damiano L, Di Stefano P, Camacho Leal MP, Barba M, Mainiero F, et al (2010) p140Cap dual regulation of E-cadherin/EGFR cross-talk and Ras signalling in tumour cell scatter and proliferation. Oncogene 29: 3677–3690. 20453886 10.1038/onc.2010.128

[8] Irby RB, Yeatman TJ (2000) Role of Src expression and activation in human cancer. Oncogene 19: 5636–5642. 11114744 10.1038/sj.onc.1203912

[9] Frame MC (2002) Src in cancer: deregulation and consequences for cell behaviour. Biochim Biophys Acta 1602: 114–130. 12020799 10.1016/s0304-419x(02)00040-9

[10] Krol J, Loedige I, Filipowicz W (2010) The widespread regulation of microRNA biogenesis, function and decay. Nat Rev Genet 11: 597–610. 20661255 10.1038/nrg2843

[11] Calin GA, Croce CM (2006) MicroRNA signatures in human cancers. Nat Rev Cancer 6: 857–866. 17060945 10.1038/nrc1997

[12] Esquela-Kerscher A, Slack FJ (2006) Oncomirs - microRNAs with a role in cancer. Nat Rev Cancer 6: 259–269. 16557279 10.1038/nrc1840

[13] Lin PY, Yu SL, Yang PC (2010) MicroRNA in lung cancer. Br J Cancer 103: 1144–1148. 20859290 10.1038/sj.bjc.6605901 PMC2967070

[14] Takamizawa J, Konishi H, Yanagisawa K, Tomida S, Osada H, et al (2004) Reduced expression of the let-7 microRNAs in human lung cancers in association with shortened postoperative survival. Cancer Res 64: 3753–3756. 15172979 10.1158/0008-5472.CAN-04-0637

[15] Hayashita Y, Osada H, Tatematsu Y, Yamada H, Yanagisawa K, et al (2005) A polycistronic microRNA cluster, miR-17-92, is overexpressed in human lung cancers and enhances cell proliferation. Cancer Res 65: 9628–9632. 16266980 10.1158/0008-5472.CAN-05-2352

[16] Liu X, Sempere LF, Ouyang H, Memoli VA, Andrew AS, et al (2010) MicroRNA-31 functions as an oncogenic microRNA in mouse and human lung cancer cells by repressing specific tumor suppressors. J Clin Invest 120: 1298–1309. 20237410 10.1172/JCI39566 PMC2846041

[17] Chen X, Guo X, Zhang H, Xiang Y, Chen J, et al (2009) Role of miR-143 targeting KRAS in colorectal tumorigenesis. Oncogene 28: 1385–1392. 19137007 10.1038/onc.2008.474

[18] Lewis BP, Shih IH, Jones-Rhoades MW, Bartel DP, Burge CB (2003) Prediction of mammalian microRNA targets. Cell 115: 787–798. 14697198 10.1016/s0092-8674(03)01018-3

[19] John B, Enright AJ, Aravin A, Tuschl T, Sander C, et al (2004) Human MicroRNA targets. PLoS Biol 2: e363. 15502875 10.1371/journal.pbio.0020363 PMC521178

[20] Krek A, Grun D, Poy MN, Wolf R, Rosenberg L, et al (2005) Combinatorial microRNA target predictions. Nat Genet 37: 495–500. 15806104 10.1038/ng1536

[21] Ambros V (2004) The functions of animal microRNAs. Nature 431: 350–355. 15372042 10.1038/nature02871

[22] Bartel DP (2004) MicroRNAs: genomics, biogenesis, mechanism, and function. Cell 116: 281–297. 14744438 10.1016/s0092-8674(04)00045-5

[23] He L, Hannon GJ (2004) MicroRNAs: small RNAs with a big role in gene regulation. Nat Rev Genet 5: 522–531. 15211354 10.1038/nrg1379

[24] Di Stefano P, Damiano L, Cabodi S, Aramu S, Tordella L, et al (2007) p140Cap protein suppresses tumour cell properties, regulating Csk and Src kinase activity. EMBO J 26: 2843–2855. 17525734 10.1038/sj.emboj.7601724 PMC1894765

[25] Wheeler DL, Iida M, Dunn EF (2009) The role of Src in solid tumors. Oncologist 14: 667–678. 19581523 10.1634/theoncologist.2009-0009 PMC3303596

[26] Summy JM, Gallick GE (2003) Src family kinases in tumor progression and metastasis. Cancer Metastasis Rev 22: 337–358. 12884910 10.1023/a:1023772912750

[27] Mazurenko NN, Kogan EA, Zborovskaya IB, Kisseljov FL (1992) Expression of pp60c-src in human small cell and non-small cell lung carcinomas. Eur J Cancer 28: 372–377. 1375484 10.1016/s0959-8049(05)80056-5

[28] Masaki T, Igarashi K, Tokuda M, Yukimasa S, Han F, et al (2003) pp60c-src activation in lung adenocarcinoma. Eur J Cancer 39: 1447–1455. 12826049 10.1016/s0959-8049(03)00276-4

[29] Kim LC, Song L, Haura EB (2009) Src kinases as therapeutic targets for cancer. Nat Rev Clin Oncol 6: 587–595. 19787002 10.1038/nrclinonc.2009.129

[30] Saini S, Majid S, Yamamura S, Tabatabai L, Suh SO, et al (2011) Regulatory Role of mir-203 in Prostate Cancer Progression and Metastasis. Clin Cancer Res 17: 5287–5298. 21159887 10.1158/1078-0432.CCR-10-2619

[31] Zhou L, Qi X, Potashkin JA, Abdul-Karim FW, Gorodeski GI (2008) MicroRNAs miR-186 and miR-150 down-regulate expression of the pro-apoptotic purinergic P2×7 receptor by activation of instability sites at the 3'-untranslated region of the gene that decrease steady-state levels of the transcript. J Biol Chem 283: 28274–28286. 18682393 10.1074/jbc.M802663200 PMC2568908

[32] Viticchie G, Lena AM, Latina A, Formosa A, Gregersen LH, et al (2011) MiR-203 controls proliferation, migration and invasive potential of prostate cancer cell lines. Cell Cycle 10: 1121–1131. 21368580 10.4161/cc.10.7.15180

[33] Bian K, Fan J, Zhang X, Yang XW, Zhu HY, et al (2012) MicroRNA-203 leads to G1 phase cell cycle arrest in laryngeal carcinoma cells by directly targeting survivin. FEBS Lett 586: 804–809. 22306317 10.1016/j.febslet.2012.01.050

[34] Wang C, Zheng X, Shen C, Shi Y (2012) MicroRNA-203 suppresses cell proliferation and migration by targeting BIRC5 and LASP1 in human triple-negative breast cancer cells. J Exp Clin Cancer Res 31: 58. 22713668 10.1186/1756-9966-31-58 PMC3585778

