# Supplementary material for: miR-203 Suppresses the Proliferation and Migration and Promotes the Apoptosis of Lung Cancer Cells by Targeting SRC
Source: PLoS One. 2014 Aug 20;9(8):e105570. doi: 10.1371/journal.pone.0105570 (PMC4139332; doi:10.1371/journal.pone.0105570)
Supplement: File S1 — Table S1, Figure S1–S5. Table S1. Clinical features of lung cancer patients; Figure S1. the expression level of SRC and miR-203 in normal lung fibroblast cell line HLF cells and lung cancer cell lines A549 cells; Figure S2. miR-203 regulates PKCalpha expression in A549 cells; Figure S3. miR-203 directly regulates SRC expression at the post-transcriptional level; Figure S4. The role of SRC in the regulation of proliferation, migration, and apoptosis of lung cancer cells; Figure S5. The apoptosis of full-serum cultured A549 cells and serum-starvation cultured A549 cells. (DOC) [file pone.0105570.s001.doc]

**File S1**

**Table S**1. Clinical features of lung cancer patients.

|  | Age | Gender | Tumor subtype | Pathological Stage | Smoking history |
| --- | --- | --- | --- | --- | --- |
| Case #1 | 58 | Male | Adenocarcinoma | IIIA | Smoker |
| Case #2 | 67 | Male | Adenocarcinoma | IIB | Smoker |
| Case #3 | 48 | Male | Squamous cell carcinoma | IIIA | Smoker |
| Case #4 | 55 | Male | Squamous cell carcinoma | IIA | Never smoker |
| Case #5 | 70 | Female | Adenocarcinoma | IA | Never smoker |
| Case #6 | 62 | Female | Adenocarcinoma | IIB | Smoker |

**Figure S1. the expression level of SRC and miR-203 in normal lung fibroblast cell line HLF cells and lung cancer cell lines A549 cells. (A and B)** Western blot analysis of the SRC protein levels in A549 cells and HLF cells. A: representative image; B: quantitative analysis. **(C)** Quantitative RT-PCR analysis of SRC mRNA levels in A549 cells and HLF cells. **(D)** Quantitative RT-PCR analysis of the miR-203 levels in A549 cells and HLF cells.* P < 0.05; ** P < 0.01.

**
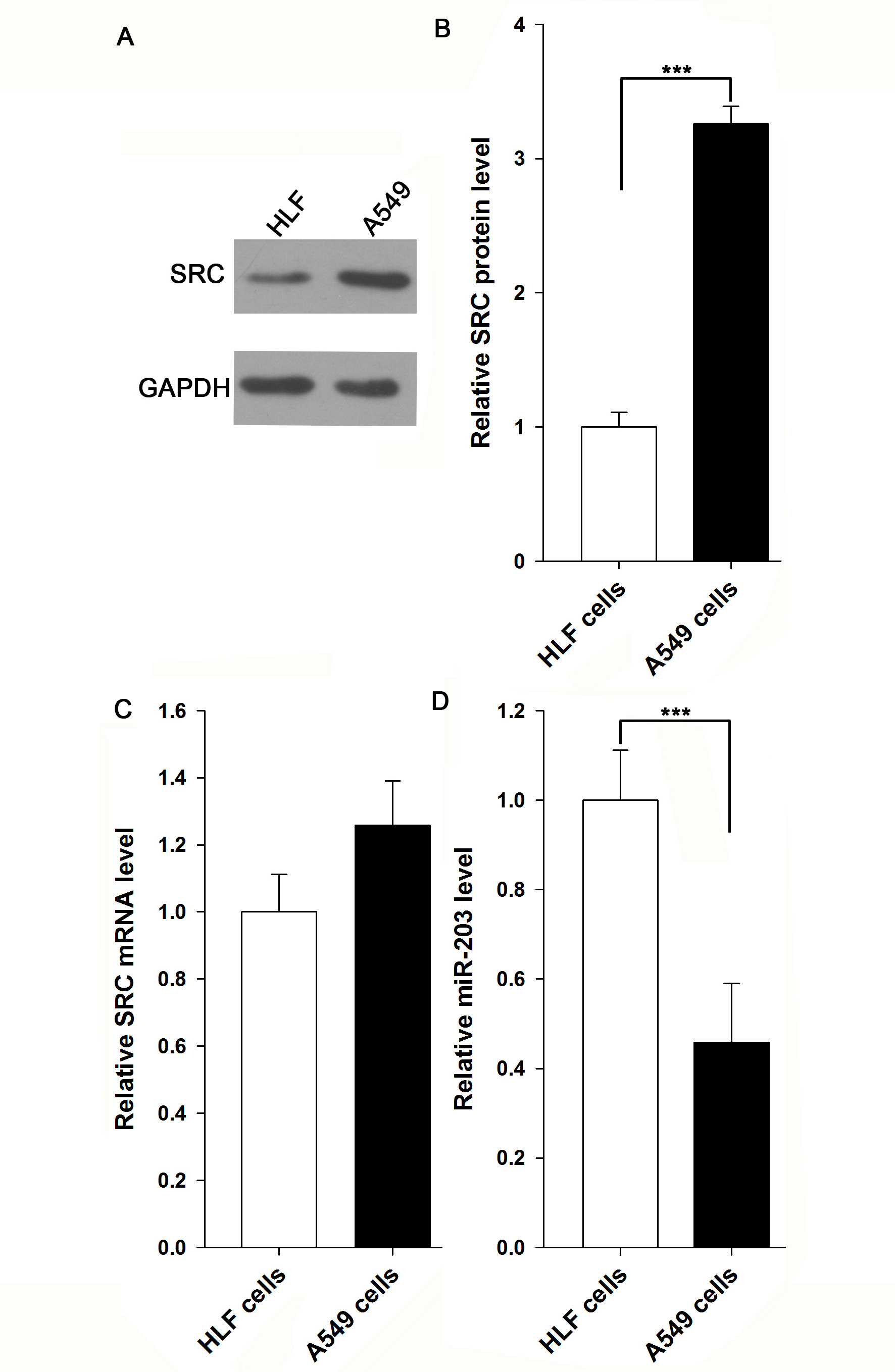
**

**Figure S2. miR-203 regulates PKCalpha expression in A549 cells.** **(A)** Quantitative RT-PCR analysis of the miR-203 levels in A549 cells treated with pre-miR-control or pre-miR-203. **(B and C)** Western blot analysis of the PKCalpha protein levels in A549 cells treated with pre-miR-control or pre-miR-203. B: representative image; C: quantitative analysis. **(D)** Quantitative RT-PCR analysis of PKCalpha mRNA levels in A549 cells treated with the pre-miR-control or pre-miR-203.

**
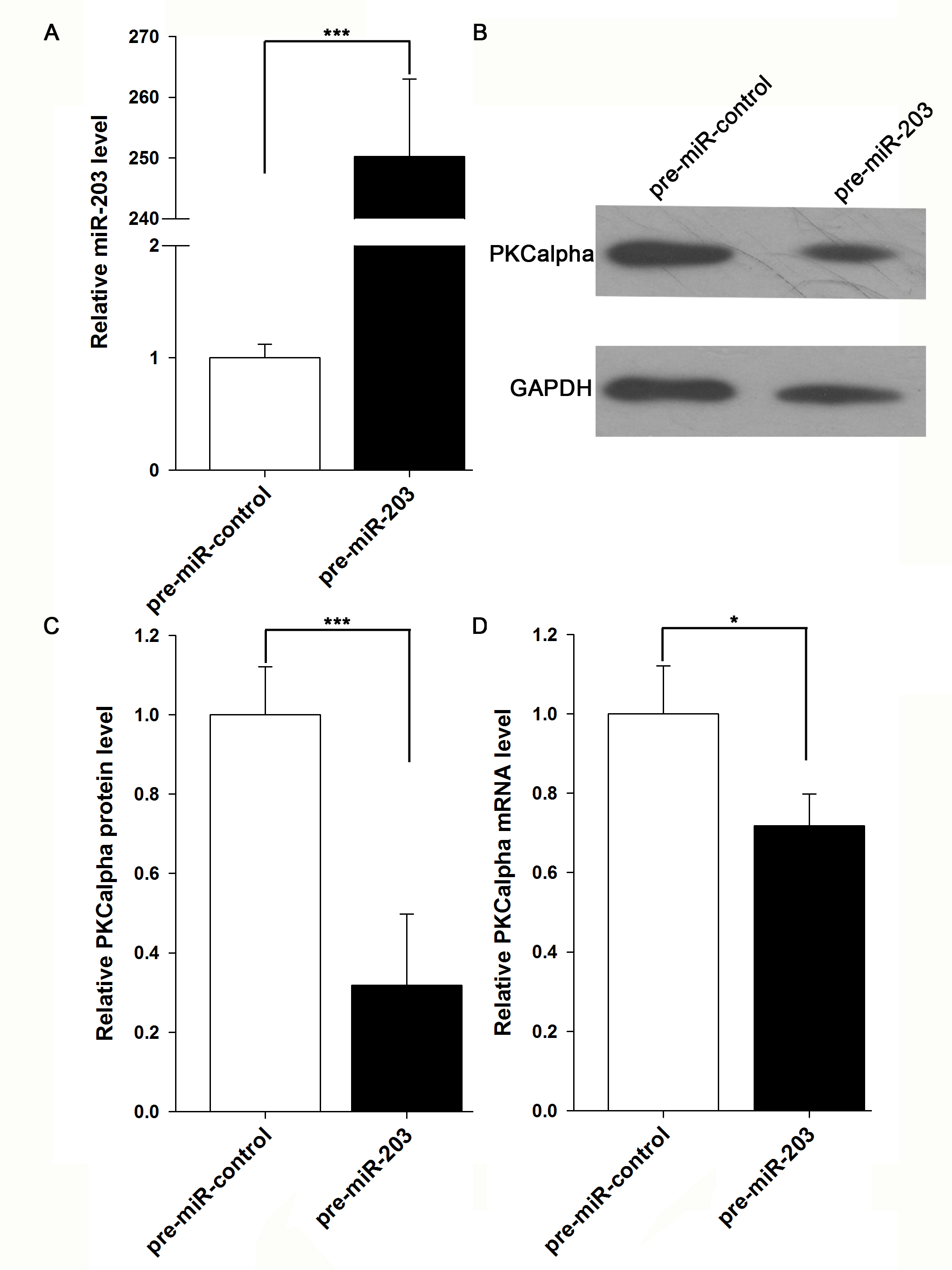
**

**Figure S3. miR-203 directly regulates SRC expression at the post-transcriptional level.** **(A)** Quantitative RT-PCR analysis of the miR-203 levels in HCC827 cells treated with pre-miR-control or pre-miR-203. **(B and C)** Western blot analysis of the SRC protein levels in HCC827 cells treated with pre-miR-control or pre-miR-203. B: representative image; C: quantitative analysis. **(D)** Quantitative RT-PCR analysis of SRC mRNA levels in HCC827 cells treated with the pre-miR-control or pre-miR-203. **(E)** Quantitative RT-PCR analysis of miR-203 levels in H1975 cells treated with the pre-miR-control or pre-miR-203. **(F and G)** Western blot analysis of the SRC protein levels in H1975 cells treated with pre-miR-control or pre-miR-203. F: representative image; G: quantitative analysis. **(H)** Quantitative RT-PCR analysis of SRC mRNA levels in H1975 cells treated with the pre-miR-control or pre-miR-203. * P < 0.05; ** P < 0.01.

**
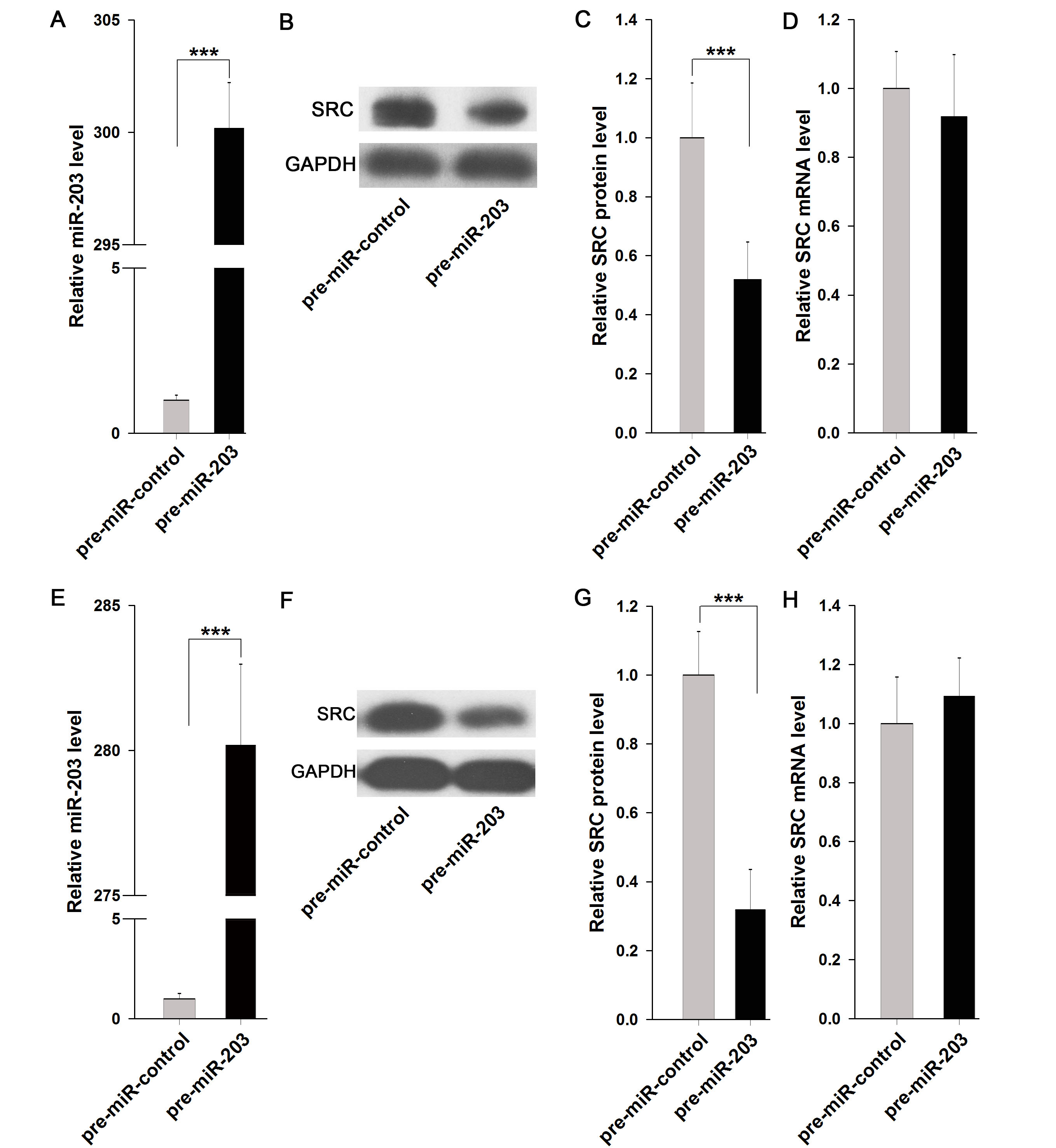
**

**Figure S4. The role of SRC in the regulation of proliferation, migration, and apoptosis of lung cancer cells. (A)** The MTT viability assay was performed 12, 24, 36, and 48 h after the transfection of A549 cells with control or SRC siRNA. **(B)** The MTT viability assay was performed 12, 24, 36, and 48 h after the transfection of A549 cells with control or SRC vector. **(C and D)** Transwell analysis of A549 cells treated with equal doses of control siRNA, SRC siRNA, control vector, or SRC vector. C: representative image; D: quantitative analysis. **(E and F)** A549 cells were transfected with equal doses of control siRNA, SRC siRNA, control vector, or SRC vector. Cell apoptosis profiles were analyzed by flow cytometry. The biparametric histogram shows cells in early (bottom right quadrant) and late apoptotic states (upper right quadrant). Viable cells are double negative (bottom left quadrant). E: representative image; F: quantitative analysis. * P < 0.05; ** P < 0.01.


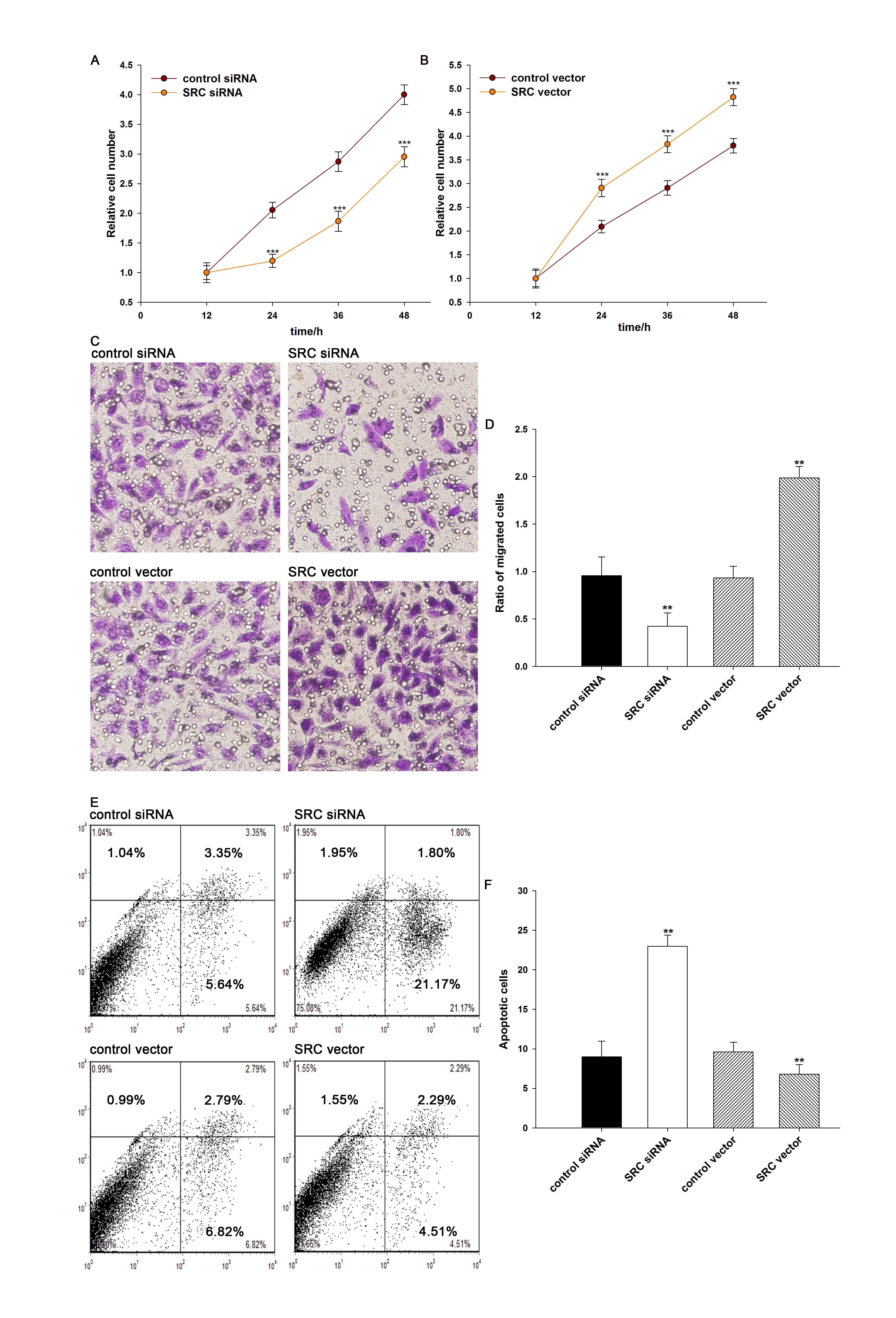


**Figure S5. The apoptosis of full-serum cultured A549 cells and serum-starvation cultured A549 cells. (A and B)** A549 cells were cultured in full-serum culture or serum-depletion culture. Cell apoptosis profiles were analyzed by flow cytometry. The biparametric histogram shows cells in early (bottom right quadrant) and late apoptotic states (upper right quadrant). Viable cells are double negative (bottom left quadrant). A: representative image; B: quantitative analysis. * P < 0.05; ** P < 0.01.

**
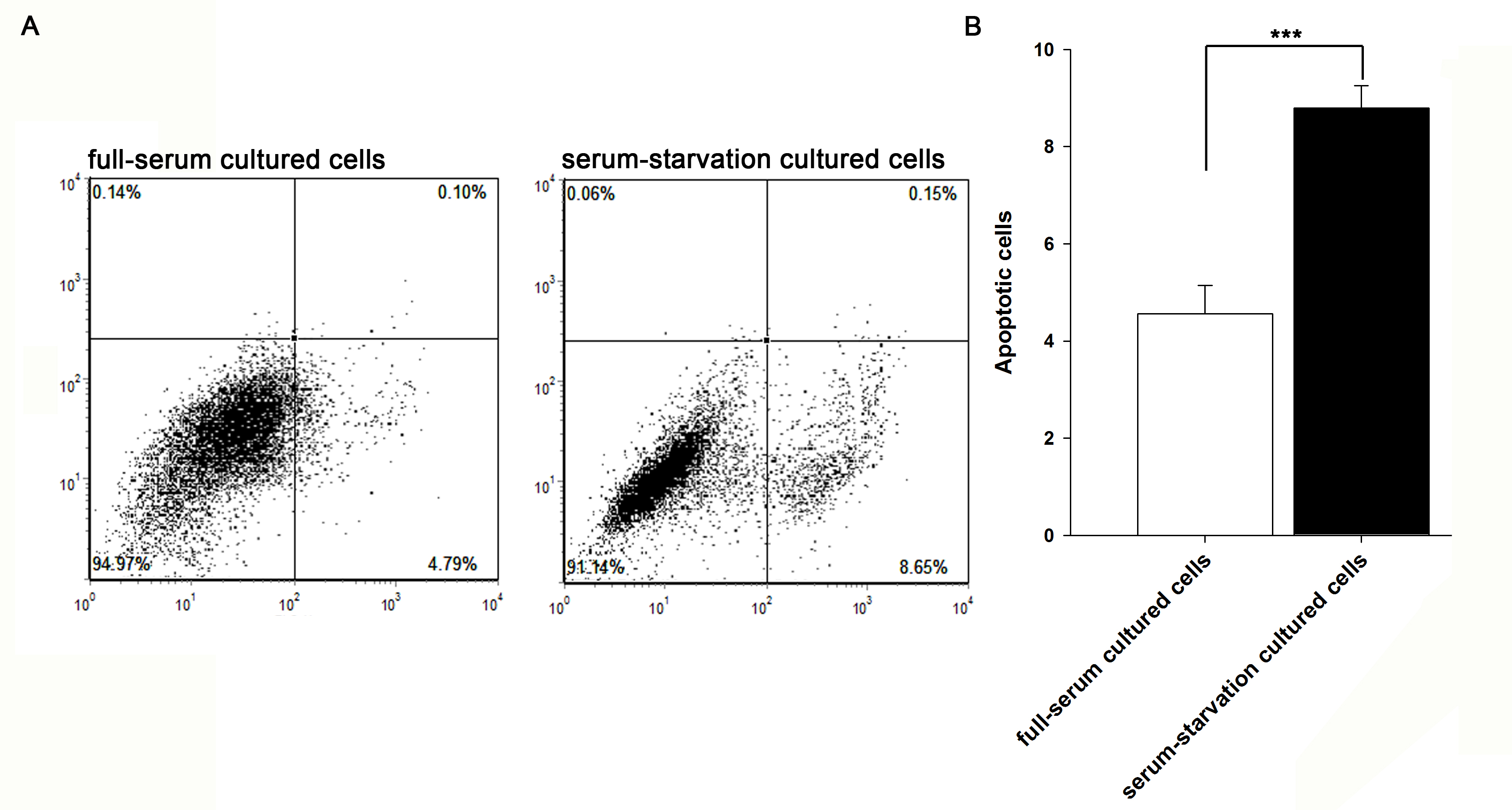
**
